# Genomic selection strategies to overcome genotype by environment interactions in biosecurity-based aquaculture breeding programs

**DOI:** 10.1186/s12711-025-00949-3

**Published:** 2025-01-22

**Authors:** Ziyi Kang, Jie Kong, Qi Li, Juan Sui, Ping Dai, Kun Luo, Xianhong Meng, Baolong Chen, Jiawang Cao, Jian Tan, Qiang Fu, Qun Xing, Sheng Luan

**Affiliations:** 1 State Key Laboratory of Mariculture Biobreeding and Sustainable Goods, Yellow Sea Fisheries Research Institute, Chinese Academy of Fishery Sciences, Qingdao, 266071 Shandong China; 2 Laboratory for Marine Fisheries Science and Food Production Processes, Qingdao Marine Science and Technology Center, Qingdao, 266237 Shandong China; 3 Ocean University of China, Fisheries College, Qingdao, 266003 China; 4 BLUP Aquabreed Co., Ltd., Weifang, 261312 China

## Abstract

**Background:**

Family-based selective breeding programs typically employ both between-family and within-family selection in aquaculture. However, these programs may exhibit a reduced genetic gain in the presence of a genotype by environment interactions (G × E) when employing biosecurity-based breeding schemes (BS), compared to non-biosecurity-based breeding schemes (NBS). Fortunately, genomic selection shows promise in improving genetic gain by taking within-family variance into account. Stochastic simulation was employed to evaluate genetic gain and G × E trends in BS for improving the body weight of *L. vannamei*, considering selective genotyping strategies for test group (TG) at a commercial farm environment (CE), the number individuals of the selection group (SG) genotyped at nucleus breeding center (NE), and varying levels of G × E.

**Results:**

The loss of genetic gain in BS ranged from 9.4 to 38.9% in pedigree-based selection and was more pronounced when G × E was stronger, as quantified by a lower genetic correlation for body weight between NE and CE. Genomic selection, particularly with selective genotyping of TG individuals with extreme performance, effectively offset the loss of genetic gain. With a genetic correlation of 0.8, genotyping 20 SG individuals in each candidate family achieved 93.2% of the genetic gain observed for NBS. However, when the genetic correlation fell below 0.5, the number of genotyped SG individuals per family had to be increased to 50 or more. Genetic gain improved by on average 9.4% when the number of genotyped SG individuals rose from 20 to 50, but the increase in genetic gain averaged only 2.4% when expanding from 50 to 80 individuals genotyped. In addition, the genetic correlation decreased by on average 0.13 over 30 generations of selection when performing BS and the genetic correlation fluctuated across generations.

**Conclusions:**

Genomic selection can effectively compensate for the loss of genetic gain in BS due to G × E. However, the number of genotyped SG individuals and the level of G × E significantly affected the extra genetic gain from genomic selection. A family-based BS selective breeding program should monitor the level of G × E and genotyping 50 SG individuals per candidate family to minimize the loss of genetic gain due to G × E, unless the level of G × E is confirmed to be low.

**Supplementary Information:**

The online version contains supplementary material available at 10.1186/s12711-025-00949-3.

## Background

Family-based selective breeding programs have achieved notable genetic gain, especially in growth-related traits in aquaculture [1, 2]. In traditional, non-biosecurity-based breeding schemes (NBS), individuals for the nucleus population (NP) are selected following rearing and testing at field-test stations [1]. However, these programs are at risk if the NP becomes infected with fatal pathogens. To mitigate this risk, a more adavanced, biosecurity-based breeding schemes (BS) have been implemented, wherein individuals from each NP family are segregated into a test group (TG) and a selection group (SG) [1], in which TG individuals are tested for target traits under diverse commercial farm environments (CE), including extensive, semi-intensive, intensive ponds, and super-intensive raceway systems, each representing unique biosecurity conditions and population densities [1, 3, 4]. Simultaneously, SG individuals are reared separately as potential selection candidates under high biosecurity levels at a nucleus breeding center (NE), with a significantly lower population density compare to CE. During the rearing process of SG, it is of utmost importance to ensure their continual preservation in a specific pathogen-free state. For example, in Pacific white shrimp (*Litopenaeus vannamei*), this involves maintaining an environment devoid of the presence of any of over ten pathogens, including white spot syndrome virus (WSSV), infectious hypodermal and haematopoietic necrosis virus (IHHNV), Vibrio parahaemolyticus causing acute hepatopancreatic necrosis disease (*Vp*_AHPND_), and others [5]. However, environmental differences between CE and NE can contribute to genotype by environment interactions (G × E), which can substantially impact genetic gains achieved through selective breeding [3].

G × E presents a significant challenge in developing superior plants and animals as it often leads to the re-ranking of genotypes across different environments, i.e., the genotype with the best phenotype within a given population in one setting may not perform as the best in another setting. This re-ranking means that the same trait measured in various environments may effectively behave like different traits [6], with the extent of re-ranking quantifiable by the genetic correlation between the trait in different environments. This phenomenon is extensively documented for aquatic species and influences many traits [4, 7–9]. For instance, genetic correlations for body weight in *L. vannamei* across various environmental conditions have been reported to ranging from 0.65 to 0.94 for different salinity levels [10], at 0.54 for various rearing densities [11], around 0.48 for different temperatures [12], and ranging from 0.17 to 0.56 for diverse culture systems [3]. Among the effects of G × E, re-ranking is particularly problematic for selective breeding programs because it can significantly hinder the desired genetic progress in CE [1, 8]. Selecting genotypes based on their phenotypes in NE may overlook those that would excel in CE. For Red tilapia (*Oreochromis spp.*), selection carried out in NE led to lower genetic gain in CE than in NE itself. Over three generations of selection, genetic gain in NE ranged from 0.01 to 1.56 genetic standard deviation units, whereas in CE it ranged from − 0.03 to 0.57 [13]. Additionally, in rainbow trout, the genetic correlation between NE and CE ranged from 0.15 to 0.48 between various environmental conditions, leading to the loss of genetic gains for body weight in CE when preselection occurred in NE [8]. Therefore, detecting and understanding the re-ranking effects of G × E is essential for strategically designing and implementing selective breeding programs.

The BS within the family-based selective breeding program of *L. vannamei* allow for selection at the family level, employing pedigree-based best linear unbiased prediction (PBLUP) for predicting estimated breeding values (EBVs) in SG. However, selecting candidates within-family based on phenotypes observed in NE may not select the individuals with the best genetics for CE, especially when G × E is strong. The limitation stems from the inability of PBLUP to estimate Mendelian sampling terms within families, which reduces the accuracy of within-family selection and genetic gain. The emergence of genomic selection has mitigated this issue by enabling more accurate estimation of genetic relationships among full-sibs and the prediction of Mendelian sampling terms [14, 15]. Critically, it provides genomic estimated breeding values (GEBVs) for genotyped SG individuals by aggregating marker effects calculated from the phenotypic and genotypic data of the genotyped TG individuals [16], thus mitigating the impact of G × E. This approach allows for more accurate selection of SG individuals, thereby enhancing the selection process in the presence of G × E.

However, genetic gain from genomic selection in BS is affected by the selective genotyping strategy (i.e., how individuals are chosen for genotyping), the number of genotyped individuals in TG and SG, and the level of G × E between NE and CE [17–23]. While much previous research has focused on biases in genomic predictions and comparative accuracies of selection among breeding schemes, there needs to be more comprehensive insight into how these factors translate into long-term genetic gain. Differences in prediction accuracy may not always reflect differences in genetic gains due to factors such as selection intensity, prediction bias, and selection-induced changes in genetic variance [21]. For species with high fecundity, like *L. vannamei*, where TG and SG comprise extensive full-sib families, it is crucial to include phenotypic data from ungenotyped individuals, employing methods such as single-step genomic BLUP (ssGBLUP) [24]. Furthermore, the optimal number of genotyped individuals within SG has not been sufficiently explored, despite its significant influence on selection intensity and associated genotyping costs [25–27]. A comprehensive understanding of how genotyping numbers affect genetic gain under varying levels of G × E and of the dynamic changes in these levels over generations is needed in order to optimize family-based selective breeding of *L. vannamei*.

Stochastic simulation is a powerful tool for the design and optimization of breeding programs, providing a fast and cost-effective method for testing alternative breeding program designs [28]. By simulating various breeding schemes, it allows breeders to identify potential challenges and optimize parameters for maximum genetic gain after long-term selection. This approach is particularly useful in complex breeding programs, where traditional trial-and-error methods would be impractical and time-consuming. Simulations have been used to improve plant breeding programs [29–31], animal breeding programs [32–34], and aquatic animal breeding programs in the presence of G × E [21, 22], as well as to address theoretical concepts in quantitative genetics and breeding [35]. In this study, we conducted simulations of a typical family-based selective breeding program for *L. vannamei* with 100 full-sib families per generation, spanning 30 generations of selection for body weight*.* The EBV for individuals in TG and SG were predicted using ssGBLUP and PBLUP under varying levels of G × E. Our research aimed to assess the loss of genetic gain in BS due to G × E, determine the optimal selective genotyping strategy within TG, investigate the optimum number of genotyped individuals in SG under different levels of G × E, and investigate the impact of selection on the level of G × E when environment conditions remain constant across generations.

## Methods

### Simulation overview

**Fig. 1:**
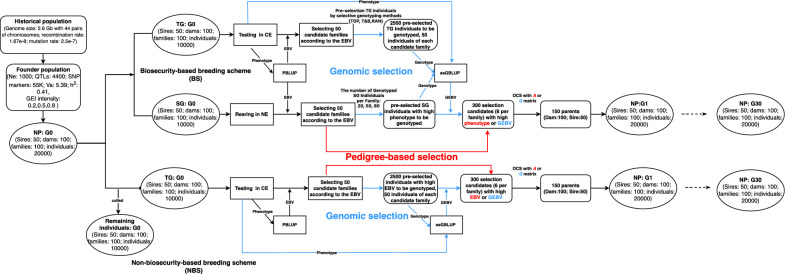
Simulation process across all breeding schemes. G × E (0.2, 0.5, 0.8): the level of genotype by environment interactions between the nucleus breeding center (NE) and the commercial farm environment (CE) in G0, quantified by the genetic correlation for body weight in the nucleus population (NP) measured in NE and CE; EBV: estimate breeding value; GEBV: genomic EBV; PBLUP: pedigree-based best linear unbiased prediction; ssGBLUP: single-step genomic BLUP; SG: selection group; RAN, TOP, and T&B: the selective genotyping strategies for individuals within each candidate family of test group (TG), involving genotyping individuals randomly, those with top-rank EBV, and those with extreme EBV, respectively; OCS: optimum contribution selection; A: pedigree-based relationship matrix; G: genomic relationship matrix; The blue lines denote the specific pathways for genomic selection, the red lines is specific to pedigree-based selection, and the black lines represent public pathways

This study conducted simulations of a family-based selective breeding program aimed at enhancing the body weight of *L. vannamei* in CE, employing both pedigree-based and genomic selection methods across 31 generations (G0 to G30) within BS and NBS. Each generation involved both between-family and within-family preselection processes. Between-family preselection was conducted using EBVs of families calculated by PBLUP. With genomic selection, within-family preselection consisted of a two-step process: initially, EBVs (for NBS) or phenotypes (for BS) were utilized to preselect individuals within each candidate family (preselected family) for genotyping. These genotyped individuals with high GEBVs, calculated using ssGBLUP, were chosen as selection candidates. In contrast, pedigree-based selection solely utilized pedigree-based EBVs and phenotypes for within-family preselection. Subsequently, optimum cross selection (OCS) was applied to optimize the mating plan [31], selecting parents from these selection candidates to generate the next generation. The breeding program design implemented in this simulation study was based on established methodologies commonly applied to *L. vannamei* [12, 36, 37]. Further details on the selection process can be found in the "Breeding Schemes" section and are illustrated in Fig. 1. Parameters for all breeding schemes are documented in Table 1.

**Table 1:** Factors for the different breeding schemes

Factors	G × E (0.2, 0.5, 0.8)			
	Genomic selection		Pedigree-based selection	
	BS	NBS	BS	NBS
NE	+	−	+	−
CE	+	+	+	+
Selection index for between-family preselection	EBV	EBV	EBV	EBV
Selection index for genotyping SG indivdiuasl with top-rank	Phenotype	−	−	−
Number of genotyping SG individuals per candidate family	20, 50, 80	−	0	−
Selection index for genotyping TG individuals	EBV	EBV	−	−
Selective genotyping strategy for TG individuals	RAN, TOP, T&B	TOP	−	−
Number of genotyped TG individuals per candidate family	50	50	0	0
Selection index for selection candidates	GEBV	GEBV	Phenotype	EBV

G × E (0.2, 0.5, 0.8): the level of genotype by environment interactions between the nucleus breeding center (NE) and the commercial farm environment (CE) in G0, quantified by the genetic correlation for body weight in the nucleus population (NP) measured in NE and CE; −: excluding; + : including; BS: biosecurity-based breeding schemes; NBS: non-biosecurity-based breeding schemes; NE: nucleus breeding center; CE: commercial farm environment; SG: selection group; TG: test group; RAN, TOP, and T&B: the selective genotyping strategies for TG individuals within each candidate family, involving genotyping individuals randomly, those with top-rank EBVs, and those with extreme EBVs, respectively

In the BS with pedigree selection, individuals from NP families were randomly divided into SG and TG, located in NE and CE, respectively. SG individuals were potential selection candidates, while TG individuals were used for sib-testing. The NBS used only TG individuals in CE for both testing and selection. In the BS with genomic selection, three selective genotyping strategies were employed for TG individuals in each preselected candidate family: RAN, TOP, and T&B, which involved genotyping 50 TG individuals randomly (RAN), the 50 individuals with the most extreme EBV (top-rank 25 and bottom-rank 25) for T&B, and the 50 individuals with top-rank EBV for TOP. Additionally, the 20, 50, or 80 SG individuals with highest phenotypes were genotyped within each preselected candidate family to represent small, medium, and large family sizes in aquaculture breeding [16, 38]. Selective genotyping of SG individuals with highest phenotypes aimed to maximize selection intensity [21], and genotyping 50 TG individuals within each candidate family was deemed sufficient for accurate GEBVs [16, 38]. Only the 50 individuals with the highest EBV in each preselected candidate family were genotyped for NBS. For the NP of G0, three levels of G × E were considered, as quantified by the genetic correlation (0.2, 0.5, and 0.8) between the body weight of NP measured in NE and CE, with lower correlations indicating stronger G × E. This range of correlations reflects the range of genetic correlation estimates for body weight of *L. vannamei* between NE and CE, which spans from 0.17 to 0.95 [3, 10–12]. Correlations above 0.8 were considered to indicate weak G × E, while those below 0.5 suggested strong G × E [1]. A total of 36 breeding schemes were analyzed, 12 for each level of G × E. Ten replicates were simulated for each breeding scheme, assessing genetic gains and changes in the level of G × E for body weight over 30 generations. The simulations were conducted using the R package AlphaSimR [28] and all procedures for GEBV and EBV calculation were performed using the BLUPF90 software [39]. OCS was performed using the AlphaMate software [40], with the Pareto optimality targeted trigonometric degree set to 45, which results in desirable genetic gain and control of inbreeding in each generation [41–43]. The source code is available at https://github.com/kzy599/Biosecurity-based-breeding-schemes.

### Simulation of genotypes and phenotypes

To simulate the genome of 1000 founders of *L. vannamei*, 44 chromosome pairs were generated, each with a genetic length of 43.41 Morgans (approximately 0.986 Morgan per chromosome) and a physical length of 2.6e9 base pairs. This was accomplished using a Markovian Coalescent Simulator based on mutation-drift equilibrium theory [44]. A recombination rate of 1.67e−8 per base pair (43.41 Morgans/2.6e9 base pairs) and a mutation rate of 2.5e−7 per base pair were simulated [45]. The effective population size was set at 1000, with historical increments to 1115 at 45 generations ago, 2651 at 122 generations ago, and 8253 at 952 generations ago, in order to reflect demographic events [36].

From the founder genomes, we randomly selected 55,000 single nucleotide polymorphisms (SNPs), with 1250 SNPs per chromosome, and an additional 4400 quantitative trait loci (QTLs), at 100 QTLs per chromosome, each with a minor allele frequency exceeding 0.05. The number of SNPs aligns with our previously developed SNP panel [46, 47]. Because body weight of *L. vannamei* is a quantitative trait [1, 38], we assumed a large number of QTLs, each exerting a small effect, also to prevent overestimation of the information content of the genomic data [48]. There was no overlap between the SNPs and QTLs. The additive effects of QTL alleles on body weight in CE and NE were determined based on a multivariate normal distribution with a mean of zero and a variance of one, with a correlation or 0.2, 0.5, or 0.8 to simulate three levels of G × E. The simulated additive QTL effects were scaled to achieve an additive genetic variance of 5.39 in the founders for each environment. Genetic values were computed as the sum of all QTL effects for an individual and phenotypes were generated by adding a residual effect drawn from a normal distribution with a mean of zero and a variance of 7.75 to achieve a heritability of 0.41. The choice of values for the additive variance and heritability were based on unpublished estimates from a nucleus breeding population comprising 416 full-sib families and 69,930 individuals under commercial farming conditions. The large numbers of families and individuals ensures reliable results, making them representative of the heritability of body weight in *L. vannamei*. In order to simplify the simulation, the same variance components were applied to traits in both the NE and CE environments within the founder population, as this study primarily focuses on the re-ranking effect of G × E, which is not influenced by variance heterogeneity. Both NE and CE phenotypes were simulated for each individual. However, the phenotype used depended on the environment (NE or CE) where the individual was reared or tested. Environments were assumed constant across generations, with no simulated environmental variance, and only additive effects were considered in the simulation.

### Breeding schemes

Breeding schemes were classified into BS, involving NE and CE, and NBS, which did not incorporate a specific NE. For all breeding schemes, the NP was established by mating 100 females with 50 males, where each male was paired with two females per generation. This mating involved either parents from the founders or those from the previous generation's NP. The mating plan was optimized to balance genetic gain and inbreeding using OCS, except for the initial pairings from the founders, which were conducted randomly. The NP consisted of 20,000 individuals, divided into 100 full-sib families, each comprising 200 individuals, which were equally divided between SG and TG by family. The phenotype of SG individuals was excluded from PBLUP or ssGBLUP, since the target trait was the body weight in the CE and to reflect scenarios encountered in practical breeding, where SG families are independently reared and are each subject to a pronounced common environment effect, which can introduce biases into the evaluation of the target trait body weight in the CE. Sex ratio was maintained at 1:1 for each populations and groups. To ensure comparability, the same populations and groups from G0 were consistently used across all breeding schemes. Furthermore, the numbers of preselected families (50) and individuals (6 per preselected family) were constant across all generations and breeding schemes to maintain consistent selection intensity.

In the BS, the top 50 families based on EBVs calculated by PBLUP were preselected to be candidate families (between-family preselection). In the pedigree-based selection, the 6 SG individuals (4 females and 2 males) with the highest phenotypes in each of the 50 candidate families were chosen as selection candidates. Genomic selection involved genotyping 50 TG individuals according to three selective genotyping strategies (RAN, TOP, T&B), and genotyping 20, 50 or 80 SG individuals with the highest phenotypes within each of the 50 candidate families. Based on the GEBVs of these genotyped SG individuals, calculated using ssGBLUP, the top 6 SG individuals (4 females and 2 males) per candidate family were chosen as selection candidates. These selection candidates were further narrowed down to 150 individuals (100 females and 50 males) used for breeding using OCS, which utilized either the A matrix from pedigree data or the G matrix from genomic data, to produced the NP of the next generation. Each male was paired with two females, and each female was paired only once, generating 100 full-sibling families.

All the procedures in NBS were identical to those in BS, except that TG individuals were used as potential selection candidates instead of SG individuals. Within-family preselection in pedigree-based NBS was based on EBVs. In genomic selection, the 50 TG individuals with the highest pedigree-based EBVs from each of the 50 candidate families were genotyped, and the 6 TG individuals (4 females and 2 males) with the highest GEBVs were chosen as selection candidates.

### Models for genetic evaluation

Considering the small effective population size of each family of *L. vannamei*, a moderate-sized training set was deemed adequate for predicting GEBVs or EBVs [16]. Moreover, a training set comprising individuals from the recent four generations can provide sufficient information to accurately estimate GEBVs or EBVs for selection candidates in the current generation [49]. Therefore, all available data on TG individuals from the last 4 generations and their parents were used to perform PBLUP or ssGBLUP to estimate the GEBV or EBV of the SG individuals in the current generation.

The GEBV or EBV of body weight of selection candidates was calculated using ssGBLUP [24] or PBLUP using the following univariate animal model, since the phenotype of SG individuals was excluded from estimation:

$${\text{y}}_{{\text{i}}} = {\upmu } + {\text{a}}_{{\text{i}}} + {\text{e}}_{{\text{i}}} ,$$

where ${\text{y}}_{\text{i}}$ denotes the phenotype of body weight of the i$\text{th}$ individual in CE; $\upmu$ denotes the overall mean; and ${\text{a}}_{\text{i}}$ and ${\text{e}}_{\text{i}}$ correspond to the additive genetic effect and the random residual effect of the the i$\text{th}$ individual at CE, respectively. These vectors of the latter were assumed to follow a multi-variate normal distribution:

$$\left[\begin{array}{c}\mathbf{a}\\ \mathbf{e}\end{array}\right]\sim N\left(\left[\begin{array}{c} \mathbf{0}\\ \mathbf{0}\end{array}\right],\right.\left.\left[\begin{array}{cc}{\mathbf{A}\upsigma }_{\text{a}}^{2}\boldsymbol{ }\text{or}\,\boldsymbol{ }{\mathbf{H}\upsigma }_{\text{a}}^{2}& \mathbf{0}\\ \mathbf{0}& {\mathbf{I}\upsigma }_{\text{e}}^{2}\end{array}\right]\right),$$

where ${\upsigma }_{\text{a}}^{2}$ and ${\upsigma }_{\text{e}}^{2}$ denote the additive genetic variance and residual variance, respectively; $\mathbf{I}$ denotes the identity matrix; **A** denotes the matrix of additive genetic relationships between individuals calculated using the pedigree information relevant for PBLUP; and **H** denotes the relationship matrix that combines the full pedigree and genomic information relevant for ssGBLUP and is expressed as [24, 39]:

$$\mathbf{H}= \left[\begin{array}{cc}{\mathbf{A}}_\mathbf{11}+{\mathbf{A}}_\mathbf{12}{\mathbf{A}}_\mathbf{22}^{-1}\left({\mathbf{G}}_{\mathbf{w}}-{\mathbf{A}}_\mathbf{22}\right){\mathbf{A}}_\mathbf{22}^\mathbf{-1}{\mathbf{A}}_\mathbf{21}& {\mathbf{A}}_\mathbf{12}{\mathbf{A}}_\mathbf{22}^\mathbf{-1}{\mathbf{G}}_{\mathbf{w}}\\ {\mathbf{G}}_{\mathbf{w}}{\mathbf{A}}_\mathbf{22}^\mathbf{-1}{\mathbf{A}}_\mathbf{21}& {\mathbf{G}}_{\mathbf{w}}\end{array}\right],$$

In which ${\mathbf{A}}_\mathbf{11}$, ${\mathbf{A}}_\mathbf{12}$, ${\mathbf{A}}_\mathbf{21}$, and ${\mathbf{A}}_\mathbf{22}$ denote the sub-matrices of **A**, and subscripts 1 and 2 represent non-genotyped and genotyped individuals, respectively; ${\mathbf{G}}_{\mathbf{w}}$ is the weighted and adjusted genomic relationship matrix to avoid singularity problems and the difference in scale and location between relationship coefficients in $\mathbf{G}$ and ${\mathbf{A}}_\mathbf{22}$ [39]:

$${\mathbf{G}}_{\mathbf{w}}{=(1-\alpha )\mathbf{G}}^{*}+\alpha {\mathbf{A}}_\mathbf{22},$$

with α was set to 0.05, and,

$${\mathbf{G}}^{\mathbf{*}}=\text{ a}+\text{b}*\mathbf{G},$$

with $\text{a}$ and $\text{b}$ inferred from the following two equations [50]:

$$(\text{Avg}.\text{diag}(\mathbf{G})*\text{b}) +\text{a}=\text{Avg}.\text{diag}({\mathbf{A}}_\mathbf{22}),$$

$$(\text{Avg}.\text{offdiag}(\mathbf{G})*\text{b}) +\text{a}=\text{Avg}.\text{offdiag}({\mathbf{A}}_\mathbf{22}),$$

where $\text{Avg}.\text{diag}$ is the average of the diagonal elements, and $\text{Avg}.\text{offdiag}$ is the average of the off-diagonal elements. The matrix $\mathbf{G}$ was computed using the first method described in [51]. Variances ${\upsigma }_{\text{a}}^{2}$ and ${\upsigma }_{\text{e}}^{2}$ were calculated using the varA() and varP() functions of AlphaSimR [28] based on the NP of the current generation, rather than the genotyped individuals, to avoid potential bias.

### Genetic gain

Each individual had two distinct genetic values, corresponding to their respective performances in two environments. For the purpose of this study, when calculating genetic gain, only the genetic values in CE were considered, which aligns with the primary objective of this selective breeding program, which was to enhance the body weight in CE. Cumulative genetic gain for each generation was computed as the difference between the average genetic value of the NP in that generation and that of the NP in G0. In this study, we focus exclusively on the genetic gain observed in G30, as it encapsulates the cumulative effects of all generations of selection. For all breeding schemes, the genetic gain in G30 is presented in the Results section, and the genetic gains for the other generations are shown in Additional file 1: Figures S1, S2. For both genomic and pedigree-based selection, the loss of genetic gain in BS compared to NBS was calculated as the difference in genetic gain between BS and NBS, dividing by the genetic gain for NBS.

### The level of G × E

The level of G** × **E, which is quantifiable as the genetic correlation between body weight evaluations in the NE and CE, plays a critical role in determining the genetic gain with BS. Given that the NP of G0 was randomly derived from the founders, it is reasonable to assume that the initial level of G** × **E in the NP of G0 mirrors that in the founders, with genetic correlations set at 0.2, 0.5, or 0.8. However, the level of G × E may vary throughout the selection process, which underscores the need to closely monitor the genetic correlation between NE and CE over generations.

As two distinct genetic values are simulated for each individual, representing their body weight in NE and CE, the correlation of these genetic values is an estimate of the genetic correlation for the NP in each generation. This approach allows for a dynamic assessment of the level of G × E, offering insights into how selective breeding program influences this interaction over generations.

### Rate of inbreeding

The rate of inbreeding (∆F) per generation was calculated using the formula [52]:

$$\Delta \text{F}=[(\overline{{\text{F} }_{\text{g}}}-\overline{{\text{F} }_{\text{g}-1}})/(1-\overline{{\text{F} }_{\text{g}-1}})]*100,$$

where ${\text{F}}_{\text{g}}$ denotes the average inbreeding coefficient of all individuals within the NP in the gth generation. Inbreeding coefficients were calculated based on pedigree analysis [53].

### Statistical analyses

Using the base R stats package [54], we conducted a three-way ANOVA and subsequent Tukey’s HSD (honestly significant difference) test to assess differences in genetic gain and inbreeding rates between selective genotyping strategies, numbers of genotyped SG individuals, and initial level of G × E in BS. Differences were deemed statistically significant at a P-value less than 0.05, and significance was further indicated by confidence interval that did not encompass zero. Differences in genetic gain between BS and NBS for each initial level of G × E were tested using T-tests, with a P-value of less than 0.05 considered significant.

## Results

### The loss of genetic gain in biosecurity-based breeding schemes

**Fig. 2:**
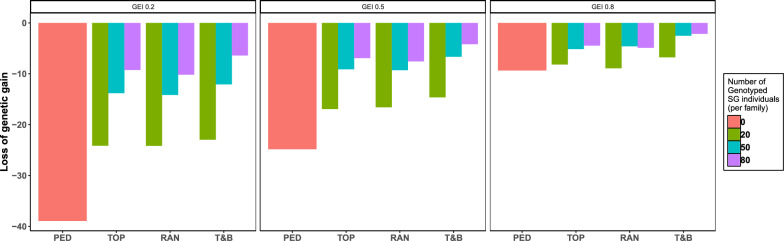
Loss of genetic gain in biosecurity-based breeding schemes(BS) when compared with non-BS (NBS). Y-axis: the percentage decrease in genetic gain relative to the NBS; PED: BS with pedigree-based selection; RAN, TOP, and T&B: the selective genotyping strategies for individuals within each candidate family of test group in BS with genomic selection, involving genotyping individuals randomly, those with top-rank EBVs, and those with extreme EBVs respectively. G × E (0.2, 0.5, 0.8): the level of genotype by environment interactions between the nucleus breeding center (NE) and the commercial farm environment (CE) in G0, quantified by the genetic correlation for body weight in the nucleus population (NP) measured in NE and CE

With pedigree-based selection (Fig. 2, Table 2), genetic gain loss for BS compared to NBS ranged from 9.4 to 38.9%, averaging 24.8%. With genomic selection, the extent of this loss depended on the level of G × E in NP of G0. For genetic correlations of 0.2 and 0.5, the genetic gain loss fluctuated between 6.4 and 24.2% (average 15.2%) and from 4.2 to 16.9% (average 10.2%), respectively, depending on the number of genotyped SG individuals in each candidate family (20, 50, or 80). A genetic correlation of 0.8 resulted in a narrower range of losses, from 2.1 to 8.9% (average 5.3%). Notably, when the genetic correlation was 0.8 and 20 SG individuals per candidate family were selected using T&B and genotyped in BS, 93.2% of the genetic gain observed in NBS was achieved. However, to attain similar gains when the genetic correlation was 0.2 or 0.5, it was necessary to genotype at least 50 SG individuals (Fig. 2, Table 2). The differences between BS and NBS (the loss of genetic gain) were all statistically significant.

**Table 2:** Genetic gain of biosecurity-based breeding schemes (BS) and non-BS (NBS) with genomic and pedigree-based selection

G × E	Number of genotyoped SG individuals	Genomic selection				Pedigree-based selection	
		BS			NBS	BS	NBS
		T&B	TOP	RAN			
0.2	0	−	−	−	85.15 $\pm$ 2.59	45.36 $\pm$ 1.99	74.27 $\pm$ 3.12
0.2	20	65.60 $\pm$ 1.97	64.59 $\pm$ 2.02	64.57 $\pm$ 2.46	−	−	−
0.2	50	74.86 $\pm 3$.18	73.38 $\pm$ 2.06	73.09 $\pm$ 2.30	−	−	−
0.2	80	79.70 $\pm$ 2.79	77.27 $\pm$ 2.82	76.48 $\pm$ 2.71	−	−	−
0.5	0	−	−	−	86.59 $\pm$ 3.12	57.16 $\pm$ 1.98	76.03 $\pm$ 2.63
0.5	20	73.91 $\pm$ 2.18	71.93 $\pm$ 2.89	72.23 $\pm$ 2.06	−	−	−
0.5	50	80.82 $\pm$ 2.30	78.69 $\pm$ 2.41	78.55 $\pm$ 2.59	−	−	−
0.5	80	82.96 $\pm$ 2.45	80.59 $\pm$ 3.48	80.04 $\pm$ 2.52	−	−	−
0.8	0	−	−	−	85.69 $\pm$ 3.19	68.45 $\pm$ 2.74	75.52 $\pm$ 3.31
0.8	20	79.89 $\pm$ 3.73	78.69 $\pm$ 3.41	78.04 $\pm$ 3.21	−	−	−
0.8	50	83.52 $\pm$ 3.81	81.28 $\pm$ 3.83	81.74 $\pm$ 2.85	−	−	−
0.8	80	83.86 $\pm$ 2.81	81.87 $\pm$ 3.31	81.49 $\pm$ 3.53	−	−	−

Genetic gain $\pm$ standard deviation; BS: biosecurity-based breeding scheme; NBS: non-biosecurity-based breeding scheme; G × E (0.2, 0.5, 0.8): the level of genotype by environment interactions between the nucleus breeding center (NE) and the commercial farm environment (CE) in G0, quantified by the genetic correlation for body weight in the nucleus population (NP) measured in NE and CE; SG: selection group; RAN, TOP, and T&B: the selective genotyping strategies for individuals within each candidate family of test group, involving genotyping individuals randomly, those with top-rank EBVs, and those with extreme EBVs, respectively

### Selective genotyping strategy within each candidate family of TG

**Fig. 3:**
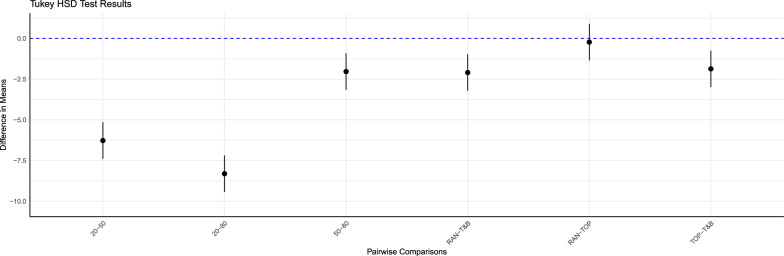
Tukey HSD test results: diferences in genetic gain by number of genotyped SG Individuals and selective genotyping strategies in TG. Black dot: the mean of the confidence interval; (20, 50, 80): the number of genotyped individuals within each candidate family of selection group (SG); RAN, TOP, and T&B: the selective genotyping strategies for individuals within each candidate family of test group (TG), involving genotyping individuals randomly, those with top-rank EBVs, and those with extreme EBVs, respectively

The best selective genotyping strategy for TG in BS was T&B (Fig. 3, Table 2), which genotyped the top 25 and bottom 25 TG individuals within each candidate family. This strategy increased genetic gain by 1.5 to 3.1% (average 2.4%) compared to TOP and by 1.6 to 4.2% (average 2.4%) compared to RAN, although these differences were not all statistically significant. Specifically, gain for T&B differed significantly from that of the other strategies (P < 0.01), whereas no significant difference in gains was observed between TOP and RAN (P > 0.8).

### The number of genotyped individuals within each candidate family of SG

With genomic selection (Fig. 3, Table 2), BS exhibited a significant increase in genetic gain when the number of genotyped SG individuals per candidate family was larger. Specifically, the increase of genetic gain ranged from 3.3 to 14.1% (average 9.4%) when the number of genotyped SG individuals per candidate family increased from 20 to 50. However, the increase was less pronounced, with a further increase from 50 to 80 SG individuals, ranging from − 0.3 to 6.5% (average 2.4%). Additionally, the benefits of genotyping more SG individuals became more pronounced when the level of G × E in the NP of G0 was greater. When the genetic correlation between NE and CE was 0.2, the genetic gain increased by 13.2 to 14.1% (average 13.6%) when the number of genotyped SG individuals increased from 20 to 50. Similarly, when the genetic correlation was 0.5 and 0.8, the increases in genetic gains ranged from 8.8 to 9.4% (average 9.4%) and from 3.3 to 4.7% (average 4.5%), respectively. When the number of genotyped SG individuals was raised from 50 to 80, the increases in genetic gain were more modest, ranging from 4.6 to 6.5% (average 5.3%), from 1.9 to 2.7% (average 2.4%), and from − 0.3 to 0.7% (average 0.4%) for genetic correlations of 0.2, 0.5, and 0.8, respectively. Pairwise comparisons of genetic gain for different numbers of genotyped SG individuals were all statistically significant (P < 0.01).

### Genomic selection vs pedigree-based selection

In BS, genomic selection notably surpassed pedigree-based selection in boosting genetic gain, while in NBS, the increment was more modest (Table 2). The increase of genetic gain by genomic selection over pedigree-based selection ranged from 14.0 to 75.7% (average 37.7%) for BS and from 13.5 to 14.7% (average 13.9%) for NBS. As the level of G × E increased, the benefits of genomic selection over pedigree-based selection in BS became more pronounced.

### Changes in the level of G × E over generations

**Fig. 4:**
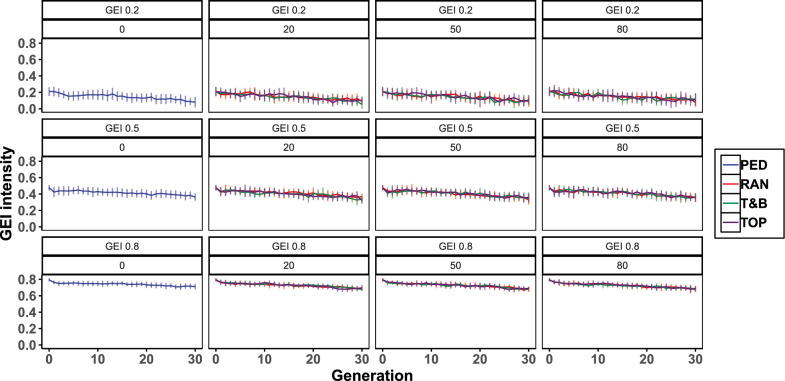
Level of G × E in nucleus population across generations for biosecurity-based breeding schemes (BS). PED: BS with pedigree-based selection; RAN, TOP, and T&B: the selective genotyping strategies for individuals within each candidate family of test group in BS with genomic selection, involving genotyping individuals randomly, those with top-rank EBVs, and those with extreme EBVs respectively; G × E (0.2, 0.5, 0.8): the level of genotype by environment interactions between the nucleus breeding center (NE) and the commercial farm environment (CE) in G0, quantified by the genetic correlation for body weight in the nucleus population (NP) measured in NE and CE; (0, 20, 50, 80): the number of genotyped individuals within each candidate family of selection group; Error bar: standard deviation

Notable changes in the level of G × E of the NP were observed over 30 generations in the BS. Specifically, the genetic correlation between NE and CE in NP of G30 showed average reductions by 0.11, 0.15, and 0.12 for initial genetic correlations of 0.2, 0.5, and 0.8 in NP of G0, respectively, signifying an increase in the level of G × E over time (Fig. 4, Table 3). Additionally, the variation in genetic correlations at G30 within the NP increased across replicates of the breeding scheme as the initial level of G × E increased. This is demonstrated by coefficients of variation of 62.5 and 75.0% under genomic selection and pedigree-based selection, respectively, for initial genetic correlation of 0.2, of 13.9 and 11.1% for 0.5, and of 4.4 and 4.2% for 0.8. This indicating greater unpredictability in genetic correlation changes at higher levels of G × E.

**Table 3:** Level of G × E in the nucleus population of G30 for biosecurity-based breeding schemes

G × E	Number of genotyped SG individuals	Genomic selection			Pedigree-based selection
		T&B	TOP	RAN	
0.2	0	–	–	–	0.08 $\pm 0.06$
0.2	20	0.05 $\pm 0.06$	0.10 $\pm 0.05$	0.09 $\pm 0.05$	–
0.2	50	0.10 $\pm 0.05$	0.11 $\pm 0.06$	0.09 $\pm 0.06$	–
0.2	80	0.11 $\pm 0.07$	0.11 $\pm 0.08$	0.08 $\pm 0.05$	–
0.5	0	–	–	–	0.36 $\pm 0.04$
0.5	20	0.34 $\pm 0.04$	0.32 $\pm 0.04$	0.37 $\pm 0.04$	–
0.5	50	0.34 $\pm 0.06$	0.36 $\pm 0.06$	0.33 $\pm 0.06$	–
0.5	80	0.37 $\pm 0.04$	0.36 $\pm 0.05$	0.36 $\pm 0.05$	–
0.8	0	–	–	–	0.71 $\pm 0.03$
0.8	20	0.68 $\pm 0.02$	0.70 $\pm 0.03$	0.69 $\pm 0.02$	–
0.8	50	0.68 $\pm 0.03$	0.69 $\pm 0.02$	0.68 $\pm 0.03$	–
0.8	80	0.67 $\pm 0.03$	0.69 $\pm 0.03$	0.68 $\pm 0.04$	–

Genetic correlation $\pm$ standard deviation; G × E (0.2, 0.5, 0.8): the level of genotype by environment interactions between the nucleus breeding center (NE) and the commercial farm environment (CE) in G0, quantified by the genetic correlation for body weight in the nucleus population (NP) measured in NE and CE; (0, 20, 50, 80): the number of genotyped individuals within each candidate family of selection group; RAN, TOP, and T&B: the selective genotyping strategies for individuals within each candidate family of test group, involving genotyping individuals randomly, those with top-rank EBVs, and those with extreme EBVs, respectively

### Rate of inbreeding

**Fig. 5:**
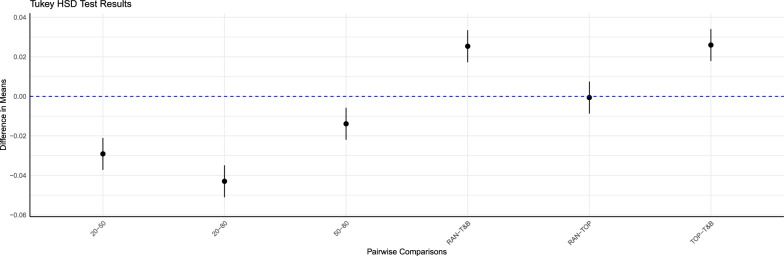
Tukey HSD test results: differences in inbreeding rate by number of genotyped SG individuals and selective genotyping strategies in TG. Black dot: the mean of the confidence interval; (20, 50, 80): the number of genotyped individuals within each candidate family of selection group (SG); RAN, TOP, and T&B: the selective genotyping strategies for individuals within each candidate family of test group (TG), involving genotyping individuals randomly, those with top-rank EBVs, and those with extreme EBVs, respectively

The inbreeding rate for all breeding schemes were less than 1% per generation, conform the Food and Agriculture Organization standards [55]. In BS with genomic selection, the inbreeding rate varied significantly depending on the number of genotyped SG individuals (Fig. 5, Table 4), with all pairwise comparisons demonstrating statistical significance (P < 0.01). The inbreeding rate was, on average, 0.59, 0.62, and 0.64% when the number of genotyped SG individuals per candidate family was 20, 50, and 80, respectively. Not all differences in the inbreeding rate between the three selective genotyping strategies were statistically significant, with significant differences observed only between T&B and other two strategies (P < 0.01), but not between TOP and RAN (P > 0.98). The inbreeding rate was, on average, 0.60% for T&B and 0.62% for both TOP and RAN.

**Table 4:** Inbreeding rate of biosecurity-based breeding schemes (BS) and non-BS (NBS) with genomic and pedigree-based selection

G × E	Number of genotyped SG individuals	Genomic selection				Pedigree-based selection	
		BS			NBS	BS	NBS
		T&B	TOP	RAN			
0.2	0	–	–	–	0.63 $\pm$ 0.03	0.69 $\pm$ 0.03	0.65 $\pm$ 0.03
0.2	20	0.58 $\pm$ 0.01	0.61 $\pm$ 0.02	0.60 $\pm$ 0.02	–	–	–
0.2	50	0.59 $\pm 0.02$	0.63 $\pm$ 0.02	0.62 $\pm$ 0.03	–	–	–
0.2	80	0.61 $\pm$ 0.01	0.65 $\pm$ 0.03	0.64 $\pm$ 0.04	–	–	–
0.5	0	–	–	–	0.63 $\pm$ 0.03	0.68 $\pm$ 0.03	0.64 $\pm$ 0.02
0.5	20	0.56 $\pm 0.02$	0.59 $\pm$ 0.02	0.59 $\pm$ 0.02	–	–	–
0.5	50	0.61 $\pm 0.02$	0.62 $\pm$ 0.03	0.62 $\pm$ 0.03	–	–	–
0.5	80	0.61 $\pm 0.02$	0.64 $\pm$ 0.02	0.64 $\pm$ 0.03	–	–	–
0.8	0	–	–	–	0.64 $\pm$ 0.03	0.67 $\pm$ 0.03	0.65 $\pm$ 0.02
0.8	20	0.57 $\pm 0.02$	0.61 $\pm$ 0.02	0.59 $\pm$ 0.02	–	–	–
0.8	50	0.60 $\pm 0.02$	0.62 $\pm$ 0.03	0.63 $\pm$ 0.03	–	–	–
0.8	80	0.62 $\pm 0.02$	0.63 $\pm$ 0.03	0.64 $\pm$ 0.02	–	–	–

Inbreeding rate $\pm$ standard deviation; BS: biosecurity-based breeding scheme; NBS: non-biosecurity-based breeding scheme; G × E (0.2, 0.5, 0.8): the level of genotype by environment interactions between the nucleus breeding center (NE) and the commercial farm environment (CE) in G0, quantified by the genetic correlation for body weight in the nucleus population (NP) measured in NE and CE; SG: selection group; RAN, TOP, and T&B: the selective genotyping strategies for individuals within each candidate family of test group, involving genotyping individuals randomly, those with top-rank EBVs, and those with extreme EBVs, respectively

## Discussion

### Loss of genetic gain in biosecurity-based breeding schemes

The loss of genetic gain when implementing BS with pedigree-based selection was mainly due to G × E between the NE and CE. In the simulated breeding schemes, the EBVs for SG individuals in the BS were calculated using between-family information. Although the within-family variance was captured through the phenotype of SG individuals, these phenotypes did not accurately represent their performance in CE [1, 8, 13, 56]. Indeed, the extent to which within-family variance was captured depended on the level of G × E between NE and CE. In contrast, in the NBS, the EBVs for TG individuals encompassed both between-family and within-family variance, as these individuals were tested in CE and also considered as potential selection candidates. With an increase in the level of G × E, the loss of genetic gain in BS due to G × E became more pronounced.

Previous research has highlighted the impact of rearing environments on the ranking of selection candidates, reporting a mean genetic correlation between environments for growth of 0.46 for rainbow trout and rohu carp, and of 0.40 for common sole (*Solea solea*). In *L. vannamei*, reported estimates of the genetic correlation for body weight between NE and CE range from 0.17 to 0.95 [3, 10–12].

Our findings indicate that genomic selection mitigated this loss of genetic gain due to G × E markedly when the genetic correlation between NE and CE was below 0.5. This advantage was likely because the GEBVs more closely represent individual performance in the CE [14, 15, 22]. The accuracy of GEBVs for growth-related traits ranged from 0.15 to 0.83, reflecting a 24% increase over pedigree-based EBV [38]. This underscores the importance of genomic selection, particularly in breeding programs that employ specific pathogen-free breeding systems where G × E between the CE and the NE exists [1]. Furthermore, as the level of G × E increased, the enhancement in genetic gain from genomic selection became more substantial, suggesting a greater return on genotyping investment with stronger level of G × E.

### Selective genotyping within each candidate family of TG

The optimal selective genotyping strategy for TG was found to be the T&B strategy, which involves genotyping individuals at the extremes of performance within each candidate family. This finding aligns with previous studies [20, 21], in which the T&B strategy was shown to enhance the accuracy of GEBVs and increase genetic gain, despite potential biases in variance estimates and in GEBVs. With GBLUP, the increases in the accuracy of GEBVs for the T&B strategy compared to the TOP strategy ranged from 15.3 to 81.0% in sheep [19], from 19.6 to 38.0% in cattle [20], and from 37.1 to 118.0% in trout [21]. Additionally, ssGBLUP further enhanced prediction accuracy and reduced biases associated with the T&B strategy [20]. The efficacy of T&B likely stems from its selective genotyping of individuals with extreme phenotypes, which greatly improves the power to detect and map QTL, as shown in various genetic association studies [57–59]. However, in our study, the differences in genetic gain between the selective genotyping strategies were not as pronounced as the differences in prediction accuracies reported in these previous studies. This may be due to the between- and within-family preselection procedures used in our study, which ensured relatively consistent selection intensity across all breeding schemes. Differences in the inbreeding rate between the selective genotyping strategies were also not as pronounced as reported in previous studies [21]. This may be because the preselection procedures in our study resulted in selection candidates to belong to 50 families for all breeding schemes.

As reported in previous studies, selective genotyping strategies may influence variance estimations and introduce bias in GEBVs calculation [18, 60, 61]. However, such bias has been shown to be minimal and does not have a significant impact on practical application [61]. Therefore, this study utilized true genetic variance in GEBVs calculation to eliminate the risk of bias and improve computational efficiency.

### The number of genotyped individuals per SG candidate family

It is not necessary to genotype all SG individuals within each candidate family. Previous studies in livestock species [25–27] showed diminishing returns when 40–60% of top-performing selection candidates were genotyped, depending on the accuracy of preselection. However, in BS for *L. vannamei*, preselection is based on the phenotypes of SG individuals and the effectiveness of phenotypic preselection is moderated by the level of G × E. The study by Chu et al. [21] on rainbow trout revealed that selecting SG individuals with the highest phenotypes within each family, even under a stronger G × E (genetic correlation as low as 0.2), can include individuals with superior performance in CE. However, this becomes increasingly challenging with a smaller number of genotyped SG individuals, particularly when the genetic correlation falls below 0.5, as it significantly limits the likelihood of including the best genotypes for CE [8]. Our findings indicated that BS can achieve most of the genetic gain of NBS by genotyping around 20 SG individuals per candidate family when the genetic correlation is 0.8. However, the required number increased to 50 SG individuals to decrease the loss of genetic gain due to G × E when the genetic correlation was below 0.5. With a genetic correlation of 0.2, genotyping more than 50 SG individuals per candidate family did not significantly improve genetic gain, since most superior genotypes were already included. These insights suggest that breeders should carefully consider G × E when determining the scale of genotyping within SG families. Additionally, increasing the number of genotyped SG individuals per family led to higher inbreeding rates because of the increase in selection intensity.

### Impact of selection on the level of G × E

G × E typically manifests in two principal ways: by causing re-ranking of selection candidates and by contributing to variance heterogeneity between environments. Genetic values of an individual in the two environments can be divided into the mean genetic effects ($\text{G}$) and the genotype by environment interaction effects ($\text{I}$) [56]. For an individual in the NE, the genetic value (${\text{GV}}_{\text{N}}$) can be modeled as $\text{G}+\text{I}$, and in the CE, the genetic value (${\text{GV}}_{\text{C}}$) can be modeled as $\text{G}-\text{I}$. Therefore, the genetic variance in NE is ${\upsigma }_{{\text{GV}}_{\text{N}}}^{2}$, = ${\upsigma }_{\text{G}}^{2}+{\upsigma }_{\text{I}}^{2}+2*\text{cov}(\text{G},\text{I})$, and in CE it is ${\upsigma }_{{\text{GV}}_{\text{C}}}^{2}$ = ${\upsigma }_{\text{G}}^{2}+{\upsigma }_{\text{I}}^{2}-2*\text{cov}(\text{G},\text{I})$. Thus, the covariance between $\text{G}$ and $\text{I}$, $\text{cov}\left(\text{G},\text{I}\right)=({\upsigma }_{{\text{GV}}_{\text{N}}}^{2}-{\upsigma }_{{\text{GV}}_{\text{C}}}^{2})/4$, captures the heterogeneity of variance between the two environments. Over generations, both ${\upsigma }_{{\text{GV}}_{\text{N}}}^{2}$ and ${\upsigma }_{{\text{GV}}_{\text{C}}}^{2}$ decreased due to selection [62]. However, the decline in ${\upsigma }_{{\text{GV}}_{\text{C}}}^{2}$ was greater (see Additional file 2: Figure S3) since selection primarily targeted genetic gain in CE. This resulted in an overall increase in $\text{cov}\left(\text{G},\text{I}\right)$ (see Additional file 3: Figure S4) and intensified variance heterogeneity. In scenarios of weak level of G × E or pedigree-based selection, $\text{cov}\left(\text{G},\text{I}\right)$ approached zero (see Additional file 3: Figure S4), indicating a reduced impact of selection on variance heterogeneity because the high genetic correlation between traits in NE and CE led to simultaneous selection for both traits. Within-family preselection with pedigree-based selection based on phenotype in NE also contributed to selection for both traits. Furthermore, $\text{cov}\left(\text{G},\text{I}\right)$ rose with the number of genotyped SG individuals, due to increased selection intensity.

The interaction variance was greater when the initial level of G × E was greater (see Additional file 3: Figure S6), as quantified by the genetic correlation, as described in the methods section.With $\text{cov}\left({\text{GV}}_{\text{N}},{\text{GV}}_{\text{C}}\right)$ =$\text{cov}\left(\text{G}+\text{I},\text{G}-\text{I}\right)={\upsigma }_{\text{G}}^{2}-{\upsigma }_{\text{I}}^{2}$, the genetic correlation is equal to$\frac{{(\upsigma }_{\text{G}}^{2}-{\upsigma }_{\text{I}}^{2})}{{\sigma }_{{\text{GV}}_{N}}{\sigma }_{{\text{GV}}_{\text{C}}}}$, which, when dividing the numerator and denominator by${\upsigma }_{\text{G}}^{2}$, becomes $\frac{1-\left(\frac{{\upsigma }_{\text{I}}^{2}}{{\upsigma }_{\text{G}}^{2}}\right)}{\frac{{\sigma }_{{\text{GV}}_{N}}{\sigma }_{{\text{GV}}_{\text{C}}}}{{\upsigma }_{\text{G}}^{2}}}$. Thus, if ${\upsigma }_{\text{I}}^{2}$ =${\upsigma }_{\text{G}}^{2}$, the genetic correlation is 0.

Over generations, ${\upsigma }_{{\text{GV}}_{\text{N}}}^{2}$ and ${\upsigma }_{{\text{GV}}_{\text{C}}}^{2}$ both trended down due to selection, reducing both ${\upsigma }_{\text{G}}^{2}$ and ${\upsigma }_{\text{I}}^{2}$ (see Additional file 3: Figures S5–S6). However, the decline in ${\upsigma }_{\text{G}}^{2}$ was faster than that in ${\upsigma }_{\text{I}}^{2}$, as the breeding program focused on enhancing genetic merit in CE rather than achieving balanced improvement across both environments [63]. This led to an increase in the ratio of ${\upsigma }_{\text{I}}^{2}$ and ${\upsigma }_{\text{G}}^{2}$ (see Additional file 3: Figure S7), indicating that the interaction variance constituted a growing proportion of the total genetic variance, subsequently leading to a decrease in the genetic correlation between NE and CE.

Throughout the selection process, the variability in the level of G × E was considerable, with high coefficients of variation observed by generation across replicates. A higher initial level of G × E, such as a genetic correlation below 0.5, was associated with a higher coefficient of variation. This suggests that with within-family selection of SG individuals—whether based on phenotype or GEBVs—leads to a substantial disparity in performance rankings of these selected individuals between NE and CE when the genetic correlation was low. These performance rankings between NE and CE were more likely to fluctuate significantly under low genetic correlation compared to high genetic correlation [1, 8, 13], resulting in a higher coefficient of variation in the level of G × E between replicates.

In practice, breeders can employ sib-testing in both environments to assess the level of G × E and determine the number of genotyped SG individuals required. However, the level of G × E also exhibited fluctuations within replicates across generations, indicating unpredictability in the trajectory of change. Such unpredictability stems from the breeding schemes prioritizing traits in CE, with the presence of G × E introducing an element of randomness into the selection of candidates. Given these observations, genotyping a large number of SG individuals (50 per candidate family) is recommended to minimize the loss of genetic gain due to G × E, unless the level of G × E is confirmed to be low. This analysis of the level of G × E in this study was purely statistical and focused solely on additive effects, suggesting that real-world complexities might pose additional challenges.

### Model of analysis

In this study, the phenotypes of SG individuals were excluded from the estimation of EBV or GEBV, and a single-trait model was used for their estimation. This approach was taken because the families of SG are reared separately in practice to prevent pathogen infection and protect the broodstocks, which may cause strong common environmental effects among full-sibs [1]. Moreover, although in practice the breeding goal may involve multiple traits [38], using information from genetically correlated traits can improve the accuracy of estimation for each trait [64]. However, multi-trait models are more challenging to converge than single-trait models. Specifically, if the number of genotyped individuals within each family of TG exceeds 60, the single-trait model has been shown to have similar predictive ability as the multi-trait model [64]. In our study, 50 TG individuals were genotyped per family and data from the last four generations were used for estimation.This resulted in prediction accuracies ranging from 0.65 $\pm$ 0.01 to 0.73 $\pm$ 0.01 for the T&B strategy, from 0.61 $\pm$ 0.02 to 0.71 $\pm$ 0.01 for TOP, and from 0.64 $\pm$ 0.02 to 0.71 $\pm$ 0.02 for RAN, indicating that the amount of information provided by genotyping 50 TG individuals per family was adequate to achieve reasonable prediction accuracy.

## Conclusions

Applying BS with genomic selection can mitigate the loss of genetic gain due to G × E, particularly when using the T&B selective genotyping strategy for the TG, which further enhances genetic gain. The number of genotyped SG individuals should be adjusted based on the level of G × E between NE and CE; the higher the level of G × E, the greater the number of genotyped individuals required. Moreover, the level of G × E tended to increase over generations of selection and its fluctuations across generations were notable. Therefore, it is advisable for breeding programs utilizing BS to monitor the level of G × E and to genotype a large number of SG individuals (50 per candidate family) to minimize the loss of genetic gain due to G × E, unless the level of G × E is confirmed to be low.

## Supplementary Information

Additional file 1: Figure S1. Genetic gains over generations within biosecurity-based breeding schemes. Figure S2. Genetic gains over generations within non-biosecurity-based breeding schemes

Additional file 2: Figure S3. Genetic variance of traits over generations in NE and CE within biosecurity-based breeding schemes

Additional file 3: Figure S4. Covariance between the mean genetic effect and the interaction effect between NE and CE within biosecurity-based breeding schemes. Figure S5. Variance of the mean genetic effect between NE and CE within biosecurity-based breeding schemes. Figure S6. Interaction variance between NE and CE within biosecurity-based breeding schemes. Figure S7. Ratio of the interaction variance to the variance of the mean genetic effect between NE and CE within biosecurity-based breeding schemes

## Data Availability

Codes are available at https://github.com/kzy599/Biosecurity-based-breeding-schemes.
