# Supplementary material for: Genomic selection strategies to overcome genotype by environment interactions in biosecurity-based aquaculture breeding programs
Source: Genet Sel Evol. 2025 Jan 22;57:2. doi: 10.1186/s12711-025-00949-3 (PMC11752716; doi:10.1186/s12711-025-00949-3)
Supplement: Supplementary file 1 — Additional file 1: Figure S1. Genetic gains over generations within biosecurity-based breeding schemes. Figure S2. Genetic gains over generations within non-biosecurity-based breeding schemes [file 12711_2025_949_MOESM1_ESM.docx]

**Additional file 1 Figure S1: Genetic gains over generations within biosecurity-based breeding schemes**

PED: biosecurity-based breeding schemes (BS) with pedigree-based selection; RAN, TOP, and T&B: the selective genotyping methods for individuals within each candidate family of test group in BS with genomic selection, involving genotyping individuals randomly, those with top-rank EBVs, and those with extreme EBVs respectively. GEI (0.2, 0.5, 0.8): the intensity of genotype by environment interaction between nucleus breeding center (NE) and commercial farm environment (CE) in G0, quantified as the genetic correlation between body weight of nucleus population measured in NE and CE; (0, 20, 50, 80): the number of genotyped individuals within each candidate family of selection group; Error bar: standard deviation.

**Additional file 1 Figure S2: Genetic gains over generations within non-biosecurity-based breeding schemes**

GEI (0.2, 0.5, 0.8): the intensity of genotype by environment interaction between nucleus breeding center (NE) and commercial farm environment (CE) in G0, quantified as the genetic correlation between body weight of nucleus population measured in NE and CE; Error bar: standard deviation.
