# Supplementary material for: Genomic selection strategies to overcome genotype by environment interactions in biosecurity-based aquaculture breeding programs
Source: Genet Sel Evol. 2025 Jan 22;57:2. doi: 10.1186/s12711-025-00949-3 (PMC11752716; doi:10.1186/s12711-025-00949-3)
Supplement: Supplementary file 2 — Additional file 2: Figure S3. Genetic variance of traits over generations in NE and CE within biosecurity-based breeding schemes [file 12711_2025_949_MOESM2_ESM.docx]

**Additional file 2 Figure S3: Genetic variance of traits over generations in NE and CE within biosecurity-based breeding schemes**

NE: nucleus breeding center; CE: commercial farm environment; PED: biosecurity-based breeding schemes (BS) with pedigree-based selection; RAN, TOP, and T&B: the selective genotyping methods for individuals within each candidate family of test group in BS with genomic selection, involving genotyping individuals randomly, those with top-rank EBVs, and those with extreme EBVs respectively. GEI (0.2, 0.5, 0.8): the intensity of genotype by environment interaction between NE and CE in G0, quantified as the genetic correlation between body weight of nucleus population measured in NE and CE; (0, 20, 50, 80): the number of genotyped individuals within each candidate family of selection group; Error bar: standard deviation.
